# The clinical relevance of physical activity education in medical school

**DOI:** 10.3402/meo.v21.30693

**Published:** 2016-01-14

**Authors:** Shaan Rashid, Omer A. Jamall, Sheeraz Iqbal, Abeer F. Rizvi, Osman Nayeem, A. M. Hameed Khan

**Affiliations:** Imperial College London, School of Medicine, London, UK

Physical activity is a vital component of good health, and it also helps reduce the severity of many chronic conditions (1). Currently, evidence suggests that physical activity counselling to patients by healthcare professionals is not being fully utilised (2). In a recent article, Dacey et al. (3) assessed the impact of medical school education on counselling knowledge and skills by future clinicians.

Although the study lacked conclusive evidence regarding the effect of medical school education and counselling knowledge/skill (because of the limited number of randomised controlled trials and homogenous outcome measures), it is a plausible assumption that doctors, having been taught how to prescribe exercise, would in turn counsel their patients effectively.

However, the article does not consider other factors, in addition to medical school education, which can have an impact on physical activity counselling by healthcare professionals.

One such factor is patient compliance. Justine et al. (4) showed that lack of time, finances, and motivation can be strong barriers to physical exercise. In addition, even if a clinician has been taught how to successfully counsel a patient, factors such as lack of consultation time may influence its implementation.

In addition, the lack of public facilities and initiatives to encourage people to exercise can also limit the amount of physical activity undertaken. From a different perspective, the organisation of the healthcare system of a country may impact the effectiveness of physical activity counselling on patients. For instance, in the United Kingdom, general practitioners (GPs) are the primary point of contact for many chronic conditions, and so educating all GPs about the effects of physical activity could have a greater impact on general health in a nation.

These wider factors lie outside the remit of counselling from the clinician and therefore the actual clinical outcomes from educating medical students regarding physical activity counselling remains unknown.

In conclusion, educating medical students about the importance of physical activity is an important aspect of the medical curricula, which is being omitted in many universities across the world. However, other factors need to be considered when assessing the clinical benefit of physical activity counselling.

*Shaan Rashid* Imperial College London School of Medicine London, UK Email: sr3011@ic.ac.uk *Omer A. Jamall* Imperial College London School of Medicine London, UK *Sheeraz Iqbal* Imperial College London School of Medicine London, UK *Abeer F. Rizvi* Imperial College London School of Medicine London, UK *Osman Nayeem* Imperial College London School of Medicine London, UK *A. M. Hameed Khan* Imperial College London School of Medicine London, UK
